# Activation of Platelet Caspase‐1 and P‐Selectin Over‐Expression in Sickle Cell Disease Patients: An Involvement of NLRP3 Inflammasome?

**DOI:** 10.1111/jcmm.71390

**Published:** 2026-10-14

**Authors:** Livia Claude, Frédéric Martino, Lydiane Mordier, Doris Célanie, Emmanuelle Bernit, Maryse Etienne‐Julan, Benoît Tressières, Marc Romana, Véronique Baccini

**Affiliations:** ^1^ INSERM, BIGR Université Paris Cité and Université des Antilles Paris France; ^2^ Laboratoire d'Hématologie CHU de la Guadeloupe Pointe‐à‐Pitre Guadeloupe; ^3^ Service de Réanimation CHU de la Guadeloupe Pointe‐à‐Pitre Guadeloupe; ^4^ Unité Transversale de la Drépanocytose CHU de la Guadeloupe, Hôpital Ricou Pointe‐à‐Pitre Guadeloupe; ^5^ Centre d'Investigation Clinique Antilles Guyane, Inserm CIC 1424 CHU de la Guadeloupe Pointe‐à‐Pitre Guadeloupe

**Keywords:** Bruton tyrosine kinase, caspase‐1, platelets, sickle cell disease

## Abstract

Sickle cell disease (SCD) is characterized by hemolytic anaemia, painful vaso‐occlusive crises, and chronic inflammation resulting partly from platelet activation. It has been shown that platelets express the nucleotide‐binding domain leucine‐rich repeat containing protein 3 (NLRP3) and the apoptosis‐associated speck‐like protein containing a CARD (ASC), forming the NLRP3 inflammasome, which controls activation of caspase‐1 and cleavage of interleukin‐1β (IL‐1β). Bruton tyrosine kinase (BTK), known to activate the NLRP3 inflammasome, is also expressed in platelets. In SCD, platelets are activated and can secrete increased quantities of IL‐1β, suggesting a possible activation of the NLRP3 inflammasome. Here, we assessed platelet P‐selectin expression, caspase‐1 activation, platelet–neutrophil aggregate formations by flow cytometry and BTK phosphorylation by Western blot in a cohort of 81 adult SCD patients at steady state condition (34 SC and 47 SS patients). We showed an increase in platelet activation in sickle patients and a high level of platelet–neutrophil aggregate in SS patients compared to healthy subjects (17 AA). We also demonstrated platelet caspase‐1 activation in SCD patients and obtained data suggesting for the first time NLRP3 inflammasome activation in SC patients. Further studies are needed to understand the mechanism of caspase‐1 activation and evaluate the potential therapeutic efficiency of Ibrutinib, a BTK inhibitor.

## 1 Introduction

Sickle cell disease (SCD) is a genetic disorder caused by the production of an abnormal haemoglobin (Hb), the so‐called haemoglobin S (HbS). HbS results from a single base mutation (A>T) at the β‐globin gene causing the substitution of valine for glutamic acid at the sixth position of the β‐globin chain. In deoxygenated conditions, HbS polymerization induces the sickling of red blood cells (RBCs) and leads to a decrease in deformability and an increase in RBCs' fragility. SCD is characterized by hemolytic anaemia, painful vaso‐occlusive crises, and chronic inflammation [1].

SCD is a generic term encompassing several sickle cell syndromes such as homozygous for the β^S^ allele or compound heterozygous resulting from the co‐inheritance of the β^S^ allele and other abnormal Hb alleles [1]. Two major sickle cell syndromes are mainly observed in the West Indies: SS and SC sickle cell patients [2]. Haemoglobin C (HbC) is the outcome of a single amino acid substitution of valine for lysine at the sixth position of the β‐globin chain. SC patients are usually described as exhibiting a moderate clinical expression of the disease compared to SS patients. However, SC patients present a hypercoagulability state and encounter complications such as retinopathy and osteonecrosis more frequently than SS patients [3, 4].

Several studies have shown that platelets are activated in SCD patients in steady state and further during crisis [5]. Platelets are known to play a critical role in haemostasis, thrombosis, and they are increasingly recognized for their role in inflammation and in innate and adaptive immune responses [6]. Indeed, activated platelets release and synthesize cytokines and chemokines [7, 8, 9], induce immune and endothelial cells' activation [10, 11, 12, 13, 14], promote the formation of aggregates with monocytes and neutrophils [15, 16] and therefore participate in vaso‐occlusion and chronic inflammation in this disease [17, 18, 19, 20].

SCD platelets secrete increased quantities of pro‐inflammatory cytokines such as interleukin‐1β (IL‐1β) [21]. IL‐1β is synthesized as a precursor protein, and its activation by cleavage requires the assemblage of a multimolecular complex called nucleotide‐binding domain leucine‐rich repeat containing protein 3 (NLRP3) inflammasome that allows caspase‐1 activation [22]. Platelets are known to express NLRP3, apoptosis‐associated speck‐like protein containing a CARD (ASC), Bruton tyrosine kinase (BTK), and caspase‐1, which are critical to forming an active inflammasome complex [23, 24]. NLRP3 inflammasome activation in platelets is known to promote platelet activation, aggregation, and thrombus formation through BTK and is reverted by BTK inhibitor [24]. In sickle cell disease, several triggers can induce platelet activation, like hemolytic damage‐associated to molecular pattern molecules (DAMPs) [25, 26, 27]. Among these DAMPs, some of them, such as haemoglobin (Hb) and heme, could lead to inflammasome NLRP3 activation in platelets. Indeed, in macrophages, heme is known to activate NLRP3 inflammasome through Toll‐like receptor 4 (TLR4) [28] which is also expressed by platelets. Hemin, an oxidized heme, induces platelet activation through PS expression and upregulation of reactive oxygen species (ROS) generation [29].

In the present study, we hypothesize that platelet caspase‐1 is activated in SC and SS patients through BTK and NLRP3 inflammasome activation. Therefore, we investigated P‐selectin expression, caspase‐1 and BTK activation in platelets and also platelet–neutrophil aggregates isolated from sickle cell patients. We also investigate platelets' in vitro activation by hemin and the effect of BTK inhibitor on this activation.

## 2 Methods

### 2.1 Human Subjects and Study Design

A total of 81 SCD patients regularly followed by the sickle cell unit (UTD) of the University Hospital of Guadeloupe were included in the study: 34 SC patients and 47 SS patients including 31 who were treated with hydroxycarbamide (HC) for more than 6 months. In addition, 17 healthy donors with the same ethnic origin were also included. All included participants were older than 18 years. SCD patients were at steady state condition at the time of inclusion, that is without acute complications (infection, vaso‐occlusive crisis (VCO), acute chest syndrome (ACS), stroke and priapism) at least 1 month before enrolment and without blood transfusions or phlebotomies in the previous 3 months. Patients and healthy control subjects were recruited after informed written consent, in accordance with the local institutional review boards (no. CNRIPH: 19‐11‐26‐67312) and the Declaration of Helsinki.

### 2.2 Standard Biological and Biochemical Variables

Cellular blood counts were obtained using XN‐1000 analyser (Sysmex, Kobe, Japan), and biochemical parameters from COBAS 4800 analyser (Roche Diagnostics, Bâle, Suisse). Fetal haemoglobin (HbF) was determined using HPLC.

### 2.3 Isolation, Washing, and Lysis of Platelets

Blood samples were collected using a 21‐gauge needle into a 3.2% trisodium citrate tube and centrifuged (170 *g*, 10 min, room temperature, brake‐free) within 1 h after collection to obtain the platelet‐rich plasma (PRP). An aliquot of PRP was used for flow cytometry, and the remaining PRP was used for protein lysates. The latter was incubated (10 min, 37°C) in the presence of prostaglandin E1 (1 μg/mL) and Apyrase (1 UI/mL), and then was centrifuged. The supernatant was discarded, and platelets were incubated (30 min, 37°C) in Tyrode albumin buffer (137 mM NaCl, 2.7 mM KCl, 12 mM NaHCO_3_, 0.42 mM NaH_2_PO_4_‐H_2_O, 1 mM MgCl_2_‐6H_2_O, 2 mM CaCl_2_‐2H_2_O, 5 mM HEPES, 0.05 g glucose, 20% bovine serum albumin, pH 7.3). Platelets were then washed and incubated in Tyrode buffer (137 mM NaCl, 0.3 mM Na_2_HPO_4_, 2 mM KCl, 12 mM NaHCO_3_, 5 mM HEPES, 5 mM dextrose, 1 mM MgCl_2_, pH 7.3) [23]. Finally, platelets were lysed with lysis buffer (10 mM Tris Base, 0.5% Triton‐X100, 25 mM NaCl, 125 mM sucrose, 25 mM NaF, 5 mM Na_4_P2O_7_‐10H_2_O, pH 7.4), 5 mM vanadate, 1 mM phenylmethylsulfonyl fluoride, 2 mM NaF, and 1/100 of protease cocktail (Sigma‐Aldrich). Proteins were stored at −20°C until used.

### 2.4 Western Blot Analysis

Phosphorylation level of BTK at Tyr223 was determined by western blot. Autophosphorylation at Tyr223 within the SH3 domain is necessary for full activation of BTK. Platelet lysates (50 μg) were separated by SDS‐PAGE and transferred to nitrocellulose membranes (Thermo Fisher Scientific, Waltham, MA, USA). The membranes were then blocked and incubated with anti‐human phosphorylated BTK (p‐BTK) (5082S; Cell Signalling Technology, Danvers, MA, USA), anti‐human BTK (3533S; Cell Signalling Technology) and anti‐human glyceraldehyde‐3‐phosphate dehydrogenase (GAPDH) (MAB374; Merck Millipore, Burlington, MA, USA). Immune complexes were revealed with secondary peroxidase‐conjugated antibodies (Dako A/S). Western blots were analysed using an Amersham Imager 600 (GE Healthcare). For quantification, densitometry was performed with ImageJ software (National Institutes of Health). The results are shown as the ratio of p‐BTK to total BTK.

### 2.5 Platelet Phenotyping

Platelet characteristics were determined by flow cytometry (Cytoflex, Beckman Coulter, Brea, CA, USA). Platelets from PRP were labelled with monoclonal antibodies (anti‐CD41‐ECD and anti‐CD62P‐APC from Beckman Coulter, Brea, CA, USA) or a green fluorescent probe called fluorescent labelled inhibitor of caspase‐1 (FLICA) that irreversibly binds to activated caspase‐1, according to the manufacturer's instructions (Immunochemistry Technologies, Davis, CA, USA). Then, platelets were washed for 5 min at 200 g with Tyrode buffer. A minimum of 15,000 events was acquired. The results were analysed with Kaluza analysis software (Beckman Coulter, Brea, CA, USA). Platelets were distinguished by specific binding of anti‐CD41 and characteristic forward and side scattering. First, platelets were gated according to size/structure (forward and side scattering) and then selected on the CD41 labeling. Anti‐CD62P antibody and FAM‐FLICA probe were used to evaluate P‐selectin surface expression and active caspase‐1, reflecting platelet and caspase‐1 activation, respectively.

### 2.6 Platelet–Neutrophil Aggregates

Characteristics of platelet–neutrophil aggregates were determined by flow cytometry (Cytoflex, Beckman Coulter, Brea, CA, USA). Whole blood was labelled with anti‐CD41‐APC (Beckman Coulter, Brea, CA, USA), anti‐CD16‐AF488 (Biolegend, San Diego, CA, USA), anti‐CD45‐ECD (Beckman Coulter, Brea, CA, USA) antibodies for 20 min at room temperature. Cells were washed for 5 min at 300 g with platelet buffer (10 mM HEPES, 145 mM NaCl, 5 mM KCl, 1 mM MgSO_4_, pH 7.4). Red blood cells were then lysed for 15 min with 1× red blood cell lysis buffer (15.5 mM NaCl, 1 mM KHCO_3_, 0.01 mM EDTA, pH 7.4) then the cells were washed with platelet buffer. This lysis step was performed twice. A minimum of 15,000 events was acquired. The results were analysed with Kaluza analysis software (Beckman Coulter, Brea, CA, USA). Platelet–neutrophil aggregates were characterized as positive events for both CD45 (panleukocyte marker), CD16 (neutrophil marker) and CD41.

### 2.7 Platelet In Vitro Stimulation by Plasma or Hemin

Platelets from healthy controls were obtained and washed like previously described. They were incubated for 1 h with plasma subjects or hemin (30 μM; Thermo Fisher), pre‐treated or not, during 10 min with a BTK inhibitor (ibrutinib, 5 μM; purchased from Selleck Chemicals, Houston, Texas). Thereafter, platelets were labelled using antibodies (anti‐CD41 ECD, anti‐CD62P APC) and a FAM‐FLICA probe.

### 2.8 Statistical Analysis

For all experiments, normality of distributions was assessed, and quantitative variables were expressed as medians and interquartile ranges (IQRs) or means and standard deviations (±SD) accordingly. Differences in patients' characteristics were assessed using the Student *t‐*test or Mann–Whitney nonparametric test, analysis of variance (ANOVA; followed by Tukey's post hoc test for multiple comparisons), or Kruskal–Wallis nonparametric test (followed by Dunn's post hoc test for multiple comparisons). Statistical analyses of flow cytometry data, western blot data were performed with ANOVA (followed by Tukey's post hoc test for multiple comparisons) or Kruskal–Wallis nonparametric test (followed by Dunn's post hoc test for multiple comparisons) according to the normality of the distributions. Pearson correlations were performed to test the associations between the parameters investigated. Statistical analyses were performed by using Prism version 8 (GraphPad Software). A *p* value < 0.05 was considered statistically significant.

## 3 Results

### 3.1 Sickle Cell Patient Cohort

Eighty‐one sickle cell patients were enrolled in our study: 34 SC patients and 47 SS patients. Biological characteristics of the study subjects at the time of blood collection are summarized in Table 1.

**TABLE 1:** Biological characteristics of studied subjects.

	AA (*n* = 17)	SC (*n* = 34)	SS (*n* = 47)
Age, median (IQR), years	52 (35–58)	40 (23–54)	47 (31–51)
HC treatment, %	NA	NA	31 (66)
Hb, mean (±SD), g/dL	13.5 (0.8)	11.1**** (1.3)	8.7**** (1.4)
HbF, median (IQR), %	1.0 (1.0–1.1)	1.5 (1.1–2.4)	11.6**** (7.1–16.1)
MCV, median (IQR), fL	91.1 (88.8–92.9)	74.5**** (65.9–80.0)	92.3 (85.5–100.0)
RET median (IQR), ×10^9^/L	56 (44–73)	109** (94–151)	209**** (141–312)
RET median (IQR), %	1.3 (1.1–1.7)	2.5* (2.0–3.5)	8.1**** (5.4–11.7)
LDH median (IQR), U/L	194.0 (180.0–227.0)	275.0 (232.0–308.5)	470.0**** (383.0–551.0)
Total bilirubin median (IQR), μM	6.0 (4.0–7.0)	18.5** (14.0–25.0)	39.0**** (26.0–72.0)
ASAT, median (IQR), U/L	20.0 (17.0–22.0)	23.0 (15.0–30.3)	36.0*** (27.0–50.0)
WBC mean (±SD), ×10^9^/L	5.9 (2.4)	7.5 (3.0)	7.7 (2.3)
PLT median (IQR), ×10^9^/L	283 (254–323)	310 (175–432)	343 (249–419)
CRP median (IQR), mg/L	0.9 (0.6–2.0)	2.8* (1.2–7.5)	4.2*** (1.8–6.9)

*Note:* *p 673 < 0.05, ** *p* < 0.01, ***p < 0.001, ****p < 0.0001.

### 3.2 Platelet Activation Is Increased in Sickle Cell Patients

We measured platelet membrane expression of CD62P (P‐selectin), a marker of platelet α granule secretion. We show that platelets are activated in SC and SS patients compared to healthy controls (Figure 1). Platelet expression of CD62P is higher in SS patients (26.86; ±SD, 11.43% vs. 13.38; ±SD, 6.64%; *p* < 0.0001) and SC patients (23.80; ±SD, 9.93% vs. 13.38; ±SD, 6.64%; *p* = 0.0026) compared to healthy controls. There was no significant difference between SC and SS patients (*p* = 0.3850). Regarding SS group, there was no significant difference between HC treated and untreated patients (27.31; ±SD, 11.54% vs. 25.99; ±SD, 11.53%; *p* = 0.7114) (Figure S1). In addition, we observed neither significant differences between patients with a history of VOC and those without, nor for patients with and without a history of ACS. The number of VOC or ACS that occurred during the 3 years preceding inclusion was not associated with platelet activation (Table S1).

**FIGURE 1:**
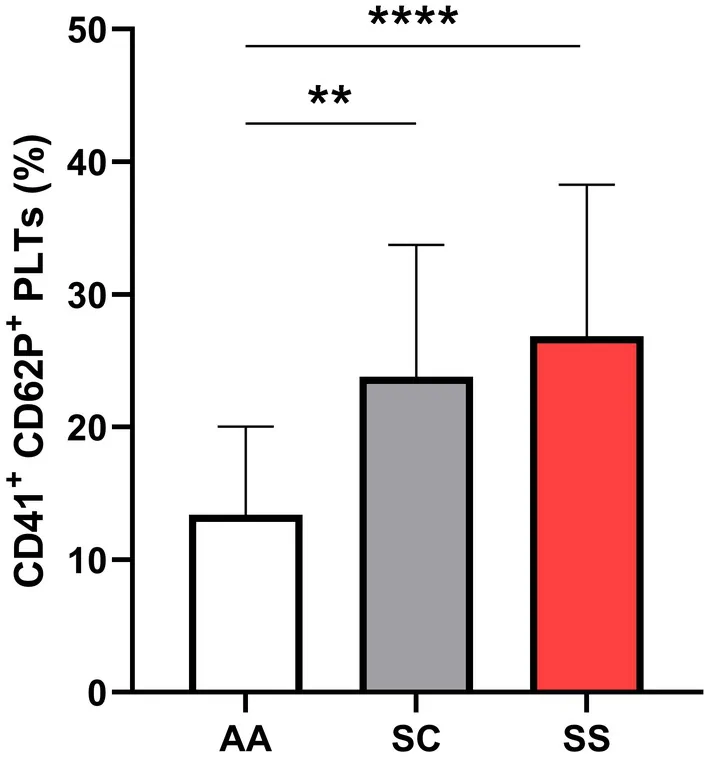
Platelet activation in sickle cell patients. Platelet P‐selectin expression (CD41^+^ CD62P^+^ PLTs) was measured by flow cytometry in 17 healthy donors, 34 SC patients and 47 SS patients. Multiple comparisons were realized between healthy subjects, SC patients and SS patients. Data are shown as mean with standard deviation. *p* values were calculated with one‐way ANOVA followed by Tukey's post hoc test for multiple comparisons. **p < 0.01, ****p < 0.0001. PLT, platelet.

### 3.3 Platelet–Neutrophil Aggregates Are Increased in SS Patients

Because platelet activation can promote the formation of platelet–neutrophil (PLT‐N) aggregates, we also assessed PLT‐N aggregates in SCD patients (Figure 2). We observed higher PLT‐N aggregates in SS patients compared to healthy subjects (16.65%; IQR, 9.58–22.23 vs. 7.18%; IQR, 5.48–12.59; *p* = 0.0056). There was no significant difference neither between SC and SS patients (9.21%; IQR, 7.06–15.85 vs. 16.65%; IQR, 9.58–22.23; *p* = 0.1144) nor between SC patients and healthy controls (9.21%; IQR, 7.06–15.85 vs. 7.18%; IQR, 5.48–12.59; *p* = 0.6574). Concerning SS group, there was no significant difference between HC treated and untreated patients (14.91; ±SD, 7.31% vs. 20.18; ±SD, 7.11%; *p* = 0.1037, Figure S2). Regarding PLT‐N aggregates, we observed neither significant differences between patients with a history of VOC and those without, nor for patients with and without a history of ACS. Furthermore, no association was found between PLT‐N aggregates and the number of VOC or ACS (Table S1).

**FIGURE 2:**
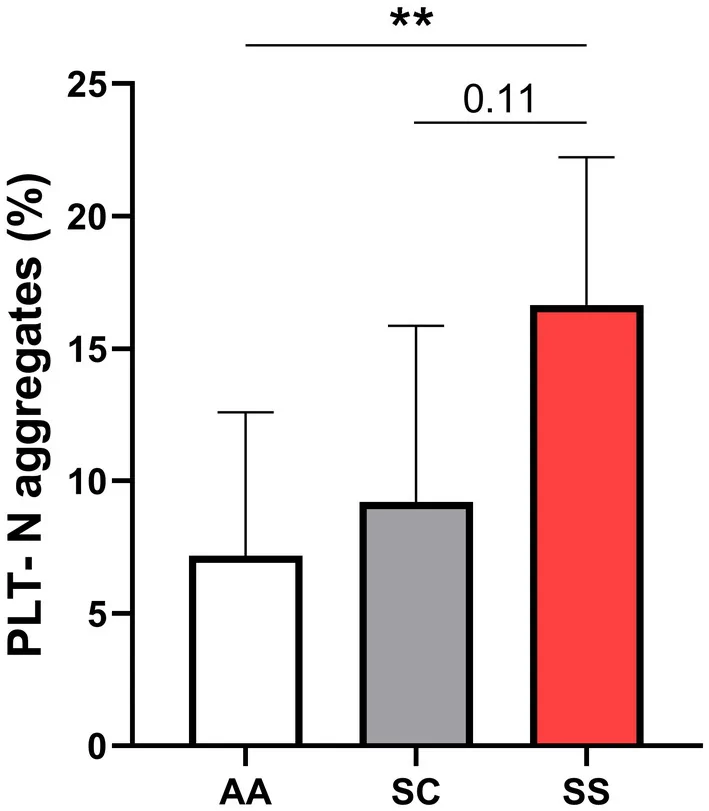
Platelet–neutrophil aggregates in sickle cell patients. Platelet–neutrophil aggregates were measured by flow cytometry in 14 healthy donors, 21 SC patients and 25 SS patients. Multiple comparisons were realized between healthy subjects, SC patients and SS patients. Data are shown as median with interquartile ranges. Inter‐group differences were assessed using the Kruskal–Wallis test followed by Dunn's post hoc test. Not significant, **p < 0.01. PLT‐N, platelet–neutrophil.

### 3.4 Activation of the Platelet Caspase‐1 Is Increased in Sickle Cell Patients and Associated With Hemolysis Markers in SS Patients

We tested the caspase‐1 activation using FAM‐FLICA probe. We have shown that, caspase‐1 is significantly more activated in SC (10%; IQR, 4.91–19.10 vs. 4.24%; IQR, 2.23–6.53; *p* = 0.0007) and SS patients (7.91%; IQR, 4.36–17.32 vs. 4.24%; IQR, 2.23–6.53; *p* = 0.0036) compared to healthy controls (Figure 3). There was no significant difference between SC and SS patients (10%; IQR, 4.91–19.10 vs. 7.91%; IQR, 4.36–17.32; *p* > 0.9999). Concerning SS group, there was no significant difference between HC treated and untreated patients (7.4%; IQR, 4.39–15.15 vs. 11.34%; IQR, 4.28–17.48; *p* = 0.5939, Figure S3). We observed a correlation between the percentage of CD41^+^ FLICA^+^ PLTs and several hemolysis markers in SS patients but not in SC patients (Table S2).

**FIGURE 3:**
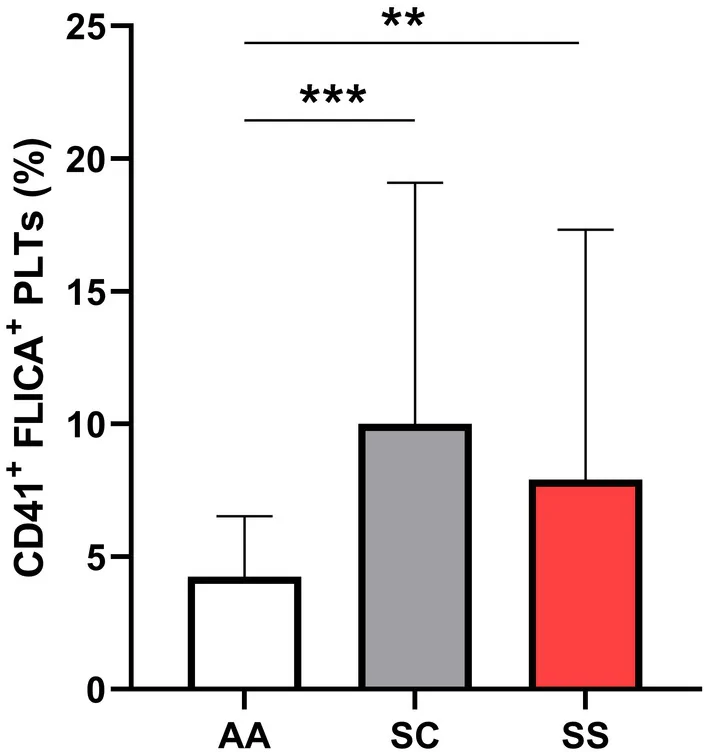
Caspase‐1 activation in platelets of sickle cell patients. Platelet caspase‐1 activation was measured by flow cytometry using FAM‐FLICA probe (CD41^+^ FLICA^+^ PLTs) in 17 healthy donors, 34 SC patients and 47 SS patients. Multiple comparisons were realized between healthy subjects, SC patients and SS patients. Data are shown as median with interquartile ranges. *p* values were calculated with Kruskal–Wallis test followed by Dunn's post hoc test. **p < 0.01, ***p < 0.001. FLICA, fluorescent labelled inhibitor of caspase‐1; PLT, platelet.

Because free haemoglobin and other markers of hemolysis such as HMGB1 are known to activate NLRP3 inflammasome and therefore caspase‐1 [28, 30, 31], we analysed the correlations between the percentage of CD41^+^ FLICA^+^ PLTs and reticulocyte rate, HbF, total bilirubin and ASAT levels. We detected that platelet caspase‐1 activation was correlated with HbF (*r* = −0.3872, *p* = 0.0216), total bilirubin (*r* = 0.3238, *p* = 0.0320) and trend to correlate with RET (*r* = 0.2825, *p* = 0.0632) and ASAT (*r* = 0.2596, *p* = 0.0889) for SS patients but not for SC patients.

We also observed a correlation between CD41^+^ FLICA^+^ PLTs and CD41^+^ CD62P^+^ PLTs percentage in SS (*r* = 0.3702, *p* = 0.0113) and SC patients (*r* = 0.4755, *p* = 0.0045, Figure S4). In addition, regarding the percentage of CD41^+^ FLICA^+^ PLTs we observed neither significant differences between patients with a history of VOC and those without, nor for patients with and without a history of ACS. The number of VOC or ACS that occurred during the 3 years preceding inclusion was not associated with platelet caspase‐1 activation (Table S1).

### 3.5 Phosphorylation of BTK in Platelets of Sickle Cell Patients

We then assessed BTK activation through p‐BTK level in platelets of healthy subjects, SC and SS patients using western blot analysis (Figure 4). We observed high rates of BTK phosphorylation in SS patients compared to healthy controls, but these results were not significant (0.35; ±SD, 0.12% vs. 0.27; ±SD, 0.11%; *p* = 0.1402). No significant difference neither between SC and SS patients (0.30; ±SD, 0.07% vs. 0.35; ±SD, 0.12%; *p* = 0.4521) nor between SC patients and healthy controls (0.30; ±SD, 0.07% vs. 0.27; ±SD, 0.11%; *p* = 0.7531) was observed. As BTK is known to activate platelet and NLRP3 inflammasome, we also assessed correlations between BTK phosphorylation, platelet and capase‐1 activation. No significant correlation between phosphorylation state of BTK and CD41^+^ FLICA^+^ PLTs percentage (*r* = 0.4262, *p* = 0.1465) and between BTK phosphorylation and CD41^+^ CD62P^+^ PLTs percentage (*r* = 0.3880, *p* = 0.1902) was found.

**FIGURE 4:**
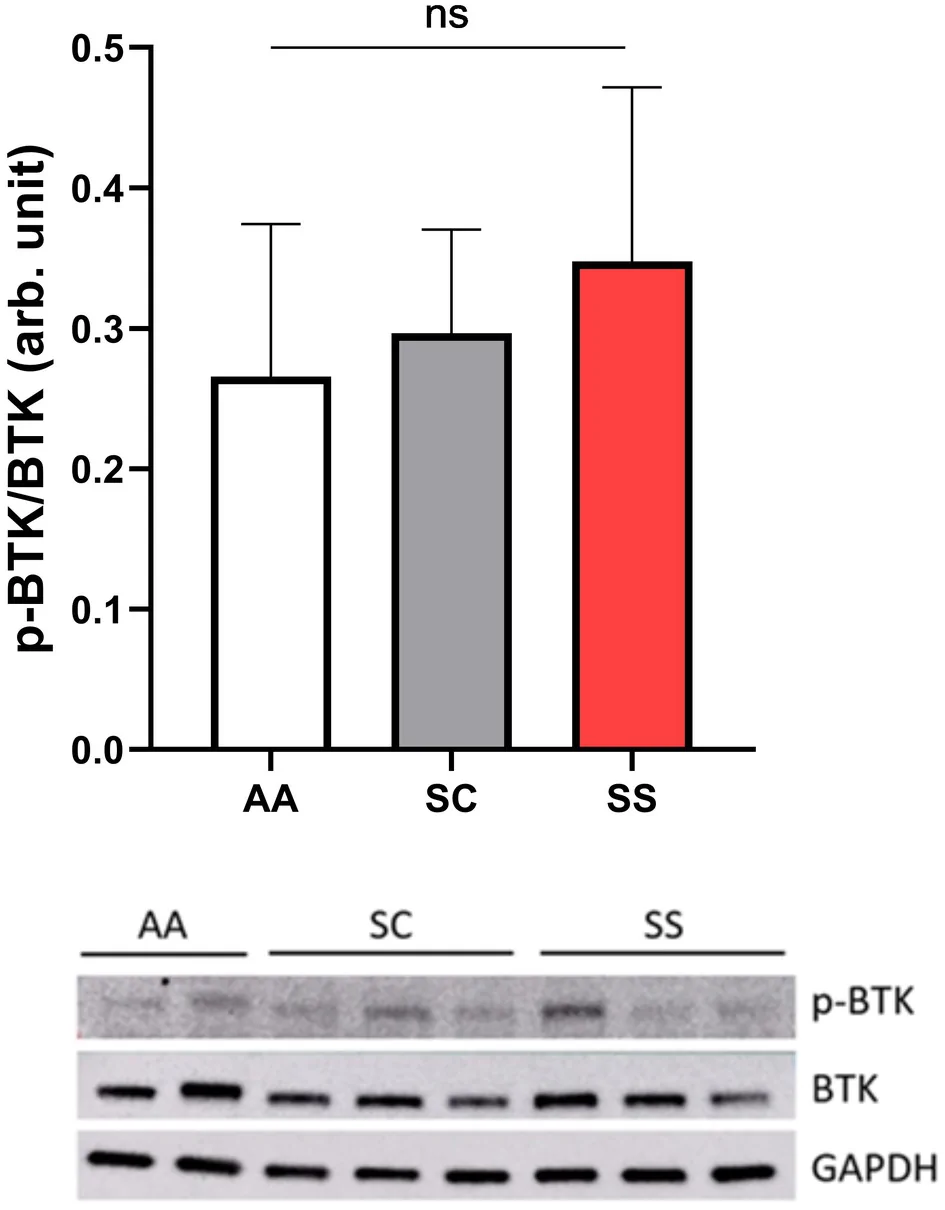
BTK phosphorylation in platelets of sickle cell patients. Phosphorylation state of BTK was measured in 12 healthy donors, 12 SC patients and 13 SS patients by the densitometric analysis of western blots and normalized to total BTK. Multiple comparisons were realized between healthy subjects, SC patients and SS patients. Data are shown as mean with standard deviation. *p* values were calculated with one‐way ANOVA followed by Tukey's post hoc test for multiple comparisons. Not significant. BTK, Bruton tyrosine kinase; p‐BTK, phosphorylated Bruton tyrosine kinase; GAPDH, glyceraldehyde‐3‐phosphate dehydrogenase.

### 3.6 Plasma From SS Patients Induces Platelet Caspase‐1 Activation

As the activation of platelet caspase‐1 is increased in SS patients and associated with hemolysis markers, we examined if plasma from SS patients could affect CD62P expression, caspase‐1 activation in platelets isolated from AA subjects. Our results showed that plasma from SS patients tends to increase platelet CD62P expression (median, 29.54%; IQR, 18.92–33.61 vs. median, 38.83; IQR, 29.66–39.47; *p* = 0.0952) (Figure 5A) and significantly increased caspase‐1 activation (median, 5.17%; IQR, 2.33–8.01 vs. median, 15.81; IQR, 13.15–38.12; *p* = 0.0286) (Figure 5B) compared to plasma from AA subjects. These data indicate that circulating factors are at least responsible for increased platelet caspase‐1 activation in SS patients.

**FIGURE 5:**
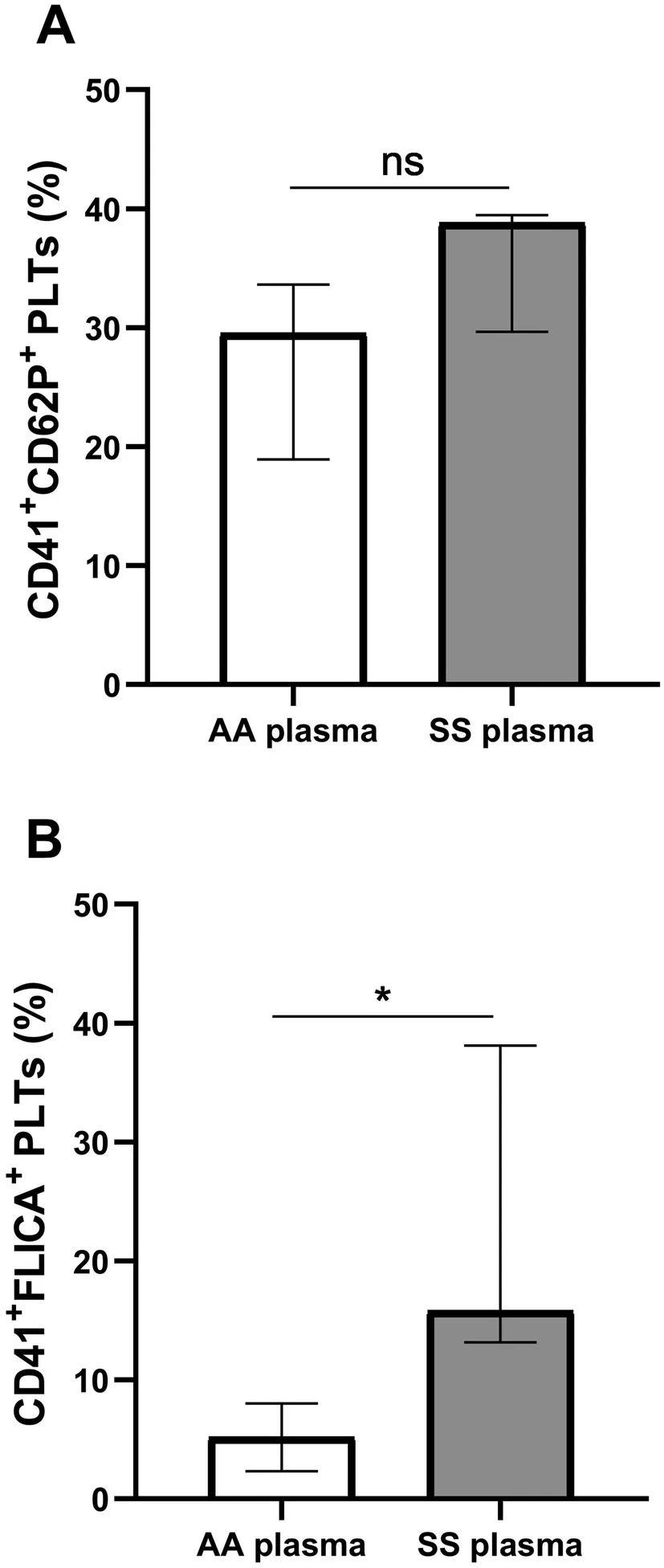
Plasma of SS patients induces platelet caspase‐1 activation. (A, B) Platelets of AA subjects were incubated with plasma from AA subjects or SS patients during 1 h. (A) P‐selectin expression and (B) caspase‐1 activations of platelets were measured by flow cytometry. Differences in the expression of P‐selectin (CD62P) and caspase‐1 (FLICA) on platelets of healthy subjects incubated with healthy subject plasma or SS plasma are illustrated in graphs A and B respectively. *p* values were calculated with the Mann–Whitney nonparametric test. Not significant, *p < 0.05. FLICA, fluorescent labelled inhibitor of caspase‐1; PLT, platelet.

### 3.7 BTK Inhibitor Ibrutinib Rescue Platelet P‐Selectin Expression Induced by Hemin but Not Totally the Caspase‐1 Activation

Then, we tested the effect of hemin, a DAMP present in the plasma of SCD patients [32, 33] and the ability of Ibrutinib, one of the available BTK inhibitors, to reverse the platelet activation induced by hemin. Our results showed that hemin significantly increased platelet CD62P expression (mean 28.78%; ±SD, 5.25 vs mean 67.21%; ±SD, 24.40; *p* = 0.0479) (Figure 6A) but not platelet caspase‐1 activation (mean 6.89%; ±SD, 4.12 vs mean 13.56%; ±SD, 2.98; *p* = 0.1221) (Figure 6B). Incubation of platelets with Ibrutinib significantly suppressed platelet CD62P expression induced by hemin (mean 67.21%; ±SD, 24.40 vs mean 10.84%; ±SD, 8.22; *p* = 0.0092) (Figure 6A), but not platelet caspase‐1 activation (mean 13.56%; ±SD, 2.98 vs mean 11.22%; ±SD, 3.18; *p* = 0.7010) (Figure 6B).

**FIGURE 6:**
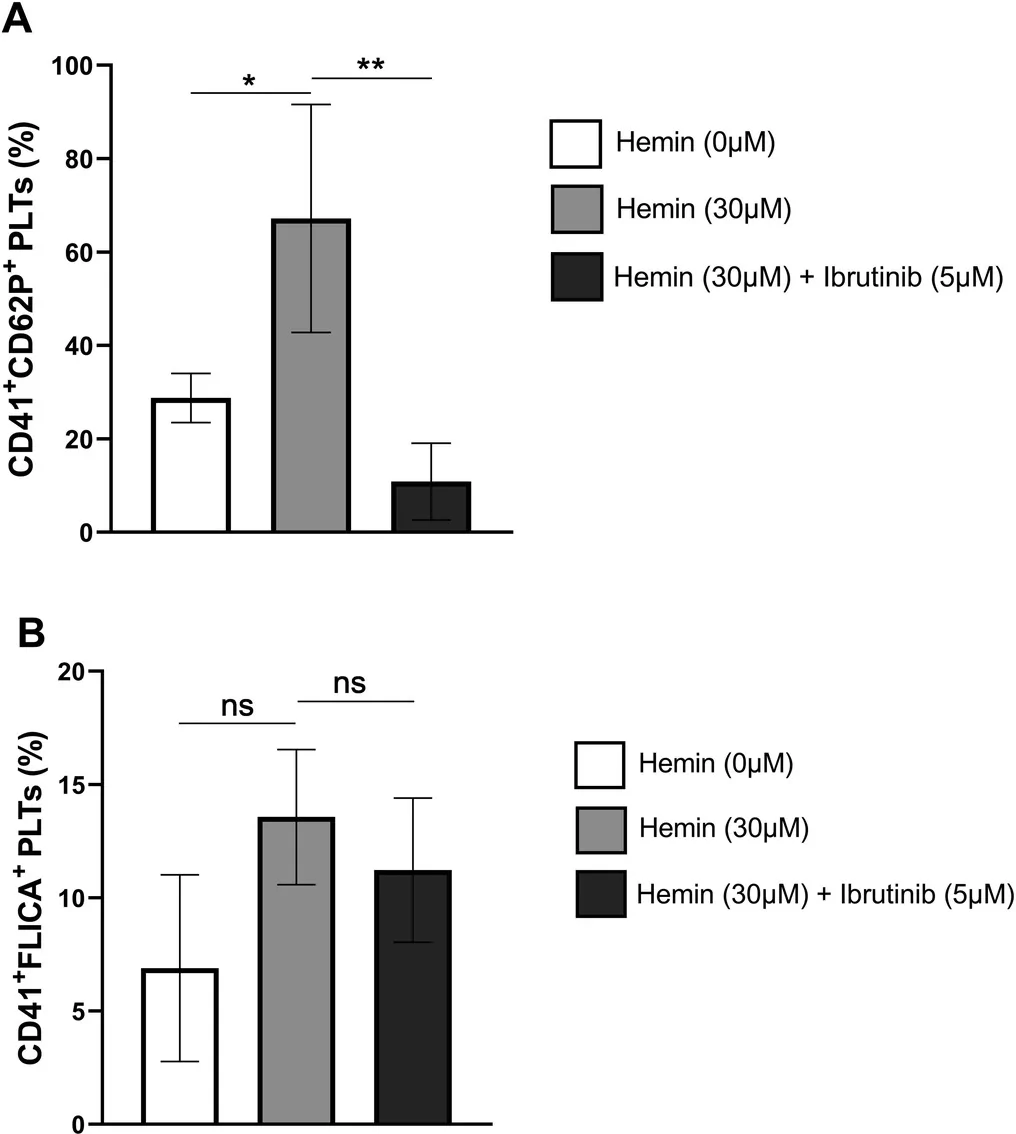
Ibrutinib inhibits platelet activation induced by hemin. (A, B) Healthy platelets were preincubated or not with 5 μM of ibrutinib during 10 min, then incubated with 30 μM of hemin during 1 h. (A) P‐selectin expression and (B) caspase‐1 activations were measured by flow cytometry. Differences in the expression of P‐selectin (CD62P) and caspase‐1 (FLICA) on healthy platelets incubated with 30 μM hemin without or with ibrutinib 5 μM are illustrated in graphs A and B respectively. *p* values were calculated with one‐way ANOVA followed by Tukey's post hoc test for multiple comparisons. Not significant, *p < 0.05, **p < 0.01. FLICA, fluorescent labelled inhibitor of caspase‐1; PLT, platelet.

These data indicate that hemin is at least responsible for increased platelet CD62P expression in SCD patients via BTK.

## 4 Discussion

In the present study, we have documented an increase in platelet activation in sickle cell patients associated with platelet caspase‐1 activation, suggesting NLRP3 inflammasome activation. We showed for the first time activation of caspase‐1 in SC patients. Moreover, we have also documented a high level of platelet–neutrophil aggregates in SS patients, and we observed a link between caspase‐1 activation and several hemolysis markers in SS patients. Then, we demonstrated in vitro that SS plasma is able to activate platelet caspase‐1 from healthy controls and that the use of Ibrutinib reverses the increase in platelet CD62P expression induced by hemin.

In sickle cell disease, activated platelets are known to promote adhesion of RBCs, monocytes, and neutrophils to the vascular endothelium [34]. Moreover, the interaction of platelets with monocytes and neutrophils induces overexpression of TF [11], promotes netosis [35] and stimulates the secretion of cytokines by these cells. All these phenomena contribute to a thromboinflammatory state resulting in vaso‐occlusion of the microcirculation [34].

Several studies described platelet activation [5, 25, 36, 37] and platelet–neutrophil aggregates formation [15, 16] in sickle cell patients. Our results confirmed platelet activation through the increase in P‐selectin surface expression in SC and SS patients.

We have also shown that SS patients have elevated levels of circulating platelet–neutrophil aggregates. It is well known that P‐selectin expression contributes to aggregate formation through the interaction of platelet P‐selectin and leukocytes PSGL‐1 [38]. Indeed, the use of a neutralizing P‐selectin antibody reduced the formation of platelet–neutrophil aggregates in mice [15, 39]. In our study, SS patients have higher platelet–neutrophil aggregates' levels compared to healthy controls that can partly explain their most severe clinical expression of the disease than SC patients. There was no significant difference between HC treated and untreated patients regarding platelet activation and platelet–neutrophil aggregates formation (Figures S1 and S2). Our latter results were in agreement with those of Villagra et al. [25] which did not show a significant difference for platelet activation between treated and untreated patients.

In our study, we have documented an activation of caspase‐1 in SC and SS patient platelets, suggesting NLRP3 inflammasome activation. To our knowledge, for the first time, we present evidence of platelet caspase‐1 activation and data suggesting an activation of the NLRP3 inflammasome in SC patients [30].

Activation of NLRP3 leads to caspase‐1 activation and then pro‐IL1β cleavage to bioactive IL‐1β [40]. It is well known now that inflammatory pathways are implicated in the propagation of the vaso‐occlusion through cellular activation [41].

A humanized monoclonal antibody targeting IL‐1β has been tested in a recent phase 2a study and showed that IL‐1β neutralization has no effect on pain but reduces leukocyte counts and high sensitivity C‐reactive protein, so reduces the pro‐inflammatory state [42].

Studies have shown that BTK is a critical activator of NLRP3 inflammasome in platelets [24], monocytes, and macrophages [43, 44]. Moreover, the NLRP3 inflammasome and BTK in platelets are known to co‐regulate platelet activation, aggregation, and in vitro thrombus formation [24]. Therefore, we evaluated the state of phosphorylation of BTK. We did not detect a significant increase in BTK phosphorylation probably due to a too limited number of patients analysed. Interestingly, we observed a negative correlation between HbF and BTK phosphorylation state (*r* = −0.5549, *p* = 0.0960) (data not shown). Another hypothesis is a lower sensitivity of western blot analysis which does not allow us to highlight a moderate increase in BTK phosphorylation.

Finally, they did not show significant correlation neither between BTK phosphorylation and platelet caspase‐1 activation nor with platelet activation.

In sickle cell disease, several triggers may be responsible for platelet activation. Indeed, hemolytic DAMPs, known to be produced at high levels in SCD patients, could be involved in such activation. Indeed, different studies demonstrated that cell‐free Hb [27, 45, 46], heme [47], HMGB1 [48] caused platelet activation. The latter is also known to induce the activation of NLRP3 inflammasome in other cell types [22, 28, 31, 49].

In this study, we showed that platelet caspase‐1 activation is associated with several hemolysis markers in SS patients but not in SC patients. Compared to SC patients, SS patients exhibited a substantial chronic hemolytic anaemia due to RBCs hemolysis, showing higher hemolysis markers levels (Hb, RET, LDH, Total bilirubin, ASAT) than the latter. Regarding caspase‐1 activation, no significant difference is observed between SC and SS patients, suggesting comparable activation of caspase‐1 in these two groups. Moreover, in these two groups, caspase‐1 activation is correlated with platelet activation. These results suggest that there could be different triggers for the two syndromes. Another explanation is that concerning SC patients, haemoglobin level is higher than SS patients with less hemolysis and less inter‐individual variability. It could explain that we do not detect any association between hemolysis markers and platelet caspase‐1 activation because our study is not statistically powerful enough.

Interestingly, we also observed a negative correlation between HbF and caspase‐1 activation (*r* = −0.3872, *p* = 0.0216) in SS patients. HbF is well known to limit the polymerization of HbS and therefore the sickling of red blood cells [50]. It is therefore tempting to hypothesize that by this mechanism, HbF decreases RBC hemolysis and therefore the release of hemolytic DAMPs. Indeed, in SS patients, HbF level was negatively correlated with reticulocyte count and total bilirubin level and positively with haemoglobin levels (data not shown).

These results suggest an indirect and beneficial effect of HbF, which would limit the phosphorylation of BTK and the activation of caspase‐1 by the decrease in hemolytic DAMPs.

Since haemolytic DAMPs are known to induce platelet CD62P expression [27, 46] and NLRP3 inflammasome activation in other cells such as macrophages [28], endothelial cells [51], we studied the effects of SS plasma and hemin on platelets from healthy subjects.

Our results showed that SS plasma tends to increase platelet activation and significantly increase platelet caspase‐1 activation. This latter result is in agreement with Vogel et al. [30] who showed that plasma from SCD patients in steady state and crisis is able to increase caspase‐1 activity in platelets from healthy subjects and that this effect is due to HMGB1.

So, we tested in vitro the effect of hemin, another hemolytic DAMP which is an iron chloride heme on healthy platelets. Plasma cell‐free heme concentrations in SCD patients are around 25 μM in steady state, so we used hemin at 30 μM [33]. We showed that hemin is able to increase platelet CD62P expression which is in accord with other studies [47, 52], which showed that hemin induces platelet activation. Naveenkumar et al. [52] have also demonstrated that hemin induces ferroptosis through ROS‐mediated proteasomal activity and inflammasome activation. Unexpectedly, in our study hemin did not provoke an increase in platelet caspase‐1 activation. This unexpected result could be related to the method used to highlight NLRP3 inflammasome activation. Hemin could not be the main hemolytic DAMP responsible for platelet caspase‐1 activation and so NLRP3 inflammasome activation in SCD patients.

Then, because BTK is known to control platelet activation [24] we studied the implication of BTK in platelet activation induced by hemin. Our results suggest that hemin induces CD62P expression through BTK. It is well known that BTK is necessary to activate PLCγ, which allows mobilization of intracellular Ca^2+^ stores, induces platelet degranulation, and therefore P‐selectin expression [53]. All of this data indicate that hemin is at least responsible for increased platelet CD62P expression in SCD patients via BTK but does not induce platelet caspase‐1 activation.

Vogel et al. [30] showed that HMGB1 induced NLRP3 inflammasome activation through TLR4 and BTK in platelets of SS patients. In this study, they also demonstrated that the use of Ibrutinib, a BTK inhibitor, currently used in onco‐haematology, significantly reverses platelet caspase‐1 activation induced by HMGB1 in sickle cell patients and SCD mice.

Another study has demonstrated that BTK inhibitor inhibits the activation of the NLRP3 inflammasome, platelet activation and aggregation, and also thrombus formation [24].

In addition to TLR4 receptor, the stimulation of other platelet receptors by hemolytic DAMPs could induce the activation of BTK/NLRP3/Caspase‐1/IL‐1β pathway in sickle cell patients. Indeed, Hb, HbS, and heme can induce platelet activation through GPIbα and C‐type‐lectin‐like receptor‐2 (CLEC‐2) respectively [27, 46, 47]. Other studies have shown that BTK plays a critical role in platelet activation mediated by GPIb and CLEC‐2 receptors [54, 55].

The activation of caspase‐1 in SCD platelets could also be due to mitochondrial ROS (Reactive oxygen species) generation induced by hemolytic DAMPs [29, 45, 52]. Indeed, mitochondrial ROS are known to provoke NLRP3 inflammasome activation in platelets [23, 52] and in other cells [28, 56, 57, 58].

Finally, a recent study has shown that the release of IL‐1β‐rich vesicles after NLRP3 inflammasome activation promoted the formation of platelet–neutrophil aggregates and provoked a lung vaso‐occlusion in SCD mice. The use of caspase‐1 inhibitor has allowed to lessen these effects [59].

BTK appears to be an interesting therapeutic target to limit thromboinflammation responsible for complications in SCD, such as stroke, venous thromboembolism, chronic inflammation, cellular interactions that trigger vaso‐occlusion, ischemia–reperfusion processes [34]. A clinical trial has started in august 2025 to evaluate the efficacy of Rilzabrutinib (a BTK inhibitor) on VOC in SCD patients [60].

In summary, our study showed platelet activation in sickle cell patients and a high level of platelet–neutrophil aggregates formation in SS patients. We also demonstrated platelet caspase‐1 activation in sickle cell patients, suggesting NLRP3 inflammasome activation. In SS patients, we presented evidence that caspase‐1 activation could result from hemolytic DAMPs. Further studies are needed to understand the mechanism of caspase‐1 activation in SC patients.

The increase in subject numbers, in particular for the evaluation of BTK phosphorylation; the study of patients in crisis, the use of inhibitors, and the stimulation of healthy platelets by hemolytic DAMPs or plasma of sickle cell patients in crisis would allow us to better understand and characterize the signalling pathways involved in platelet activation and vaso‐occlusion.

## Author Contributions

**Livia Claude:** writing – original draft, formal analysis, data curation, validation. **Frédéric Martino:** formal analysis. **Lydiane Mordier:** formal analysis. **Doris Célanie:** formal analysis. **Emmanuelle Bernit:** resources. **Maryse Etienne‐Julan:** resources. **Benoît Tressières:** methodology, software. **Marc Romana:** formal analysis. **Véronique Baccini:** conceptualization, investigation, funding acquisition, writing – original draft, formal analysis, supervision, data curation, validation.

## Funding

This work was supported by the University Hospital of Guadeloupe (Grant/Award Number: IDRCB: 2019‐A03033‐54).

## Ethics Statement

This study was approved by the Committee for the Protection of Persons in South‐East III of France (CPP Sud Est III) in accordance with the Declaration of Helsinki (no. CNRIPH: 19‐11‐26‐67312).

## Consent

All patients and healthy subjects provided verbal and written consent for participation in this study.

## Conflicts of Interest

The authors declare no conflicts of interest.

## Supporting information


**Figure S1:** Platelet activation in SS patients treated and untreated with hydroxycarbamide. Platelet P‐selectin expression (CD41^+^ CD62P^+^ PLTs) was measured by flow cytometry in 47 SS patients (16 SS and 31 HC treated SS patients). Comparison was realized between HC treated and untreated SS patients. Data are shown as mean with standard deviation. *p* value was calculated with the Student *t* test. Not significant. PLT, platelet.
**Figure S2:** Platelet–neutrophil aggregates in HC treated and untreated SS patients. Platelet–neutrophil aggregates were measured by flow cytometry in 25 SS patients (8 SS and 17 HC treated SS patients). Comparison was realized between HC treated and untreated SS patients. Data are shown as mean with standard deviation. *p* value was calculated with the Student *t* test. Not significant. PLT‐N, platelet–neutrophil.
**Figure S3:** Caspase‐1 activation in HC treated and untreated SS patients. Platelet caspase‐1 activation was measured by flow cytometry using FAM‐FLICA probe (CD41^+^ FLICA^+^ PLTs) 47 SS patients (16 SS and 31 HC treated SS patients). Comparison was realized between HC treated and untreated SS patients. Data are shown as median with interquartile ranges. *p* value was calculated with Mann–Whitney nonparametric test. Not significant. FLICA, fluorescent labelled inhibitor of caspase‐1; PLT, platelet.
**Figure S4:** Platelet activation in SS and SC patients correlate with platelet caspase‐1 activation. Positive correlations were observed between CD41^+^ CD62P^+^ PLTs and CD41^+^ FLICA^+^ PLTs percentage in (A) SS (*r* = 0.3702, *p* = 0.0113) and (B) SC patients (*r* = 0.4755, *p* = 0.0045, A, B) Pearson's correlation coefficients (*r*) were calculated and are shown in the panels. *p < 0.05, **p < 0.01. FLICA, fluorescent labelled inhibitor of caspase‐1; PLT, platelet.
**Table S1:** Clinical characteristics and platelet activation markers of studied patients. Data are median (IQR), mean (±SD). ACS, acute chest syndrome; IQR, interquartile range; SD, standard deviation; VOC, vaso‐occlusive crisis.
**Table S2:** Correlation between rate of CD41^+^ FLICA^+^ platelets and hemolysis/anaemia markers in sickle cell patients. Correlations were estimated by Pearson's correlation coefficients (*r*) in sickle cell patients. ASAT, aspartate aminotransferase; CI, confidence interval; FLICA, fluorescent labelled inhibitor of caspase‐1; RET, reticulocyte, HbF, fetal haemoglobin; PLT, platelet. Significant *p* values are in bold.

## Data Availability

The data that support the findings of this study are available from the corresponding author upon reasonable request.

## References

[1] D. C. Rees , T. N. Williams , and M. T. Gladwin , “Sickle‐Cell Disease,” Lancet 376, no. 9757 (2010): 2018–2031, 10.1016/S0140-6736(10)61029-X. 21131035

[2] C. Saint‐Martin , M. Romana , A. Bibrac , et al., “Universal Newborn Screening for Haemoglobinopathies in Guadeloupe (French West Indies): A 27‐Year Experience,” Journal of Medical Screening 20, no. 4 (2013): 177–182, 10.1177/0969141313507919. 24144845

[3] F. Lionnet , N. Hammoudi , K. S. Stojanovic , et al., “Hemoglobin Sickle Cell Disease Complications: A Clinical Study of 179 Cases,” Haematologica 97, no. 8 (2012): 8, 10.3324/haematol.2011.055202. PMC3409809 22315500

[4] M. P. Colella , E. V. de Paula , J. A. Machado‐Neto , et al., “Elevated Hypercoagulability Markers in Hemoglobin SC Disease,” Haematologica 100, no. 4 (2015): 466–471, 10.3324/haematol.2014.114587. PMC4380719 25596272

[5] T. Wun , T. Paglieroni , A. Rangaswami , et al., “Platelet Activation in Patients With Sickle Cell Disease,” British Journal of Haematology 100, no. 4 (1998): 4, 10.1046/j.1365-2141.1998.00627.x. 9531343

[6] C. N. Morrell , A. A. Aggrey , L. M. Chapman , and K. L. Modjeski , “Emerging Roles for Platelets as Immune and Inflammatory Cells,” Blood 123, no. 18 (2014): 18, 10.1182/blood-2013-11-462432. PMC4007605 24585776

[7] S. Lindemann , N. D. Tolley , D. A. Dixon , et al., “Activated Platelets Mediate Inflammatory Signaling by Regulated Interleukin 1beta Synthesis,” Journal of Cell Biology 154, no. 3 (2001): 485–490, 10.1083/jcb.200105058. PMC2196422 11489912

[8] M. M. Denis , N. D. Tolley , M. Bunting , et al., “Escaping the Nuclear Confines: Signal‐Dependent Pre‐mRNA Splicing in Anucleate Platelets,” Cell 122, no. 3 (2005): 379–391, 10.1016/j.cell.2005.06.015. PMC4401993 16096058

[9] C. N. Jenne , R. Urrutia , and P. Kubes , “Platelets: Bridging Hemostasis, Inflammation, and Immunity,” International Journal of Laboratory Hematology 35, no. 3 (2013): 254–261, 10.1111/ijlh.12084. 23590652

[10] V. Henn , J. R. Slupsky , M. Gräfe , et al., “CD40 Ligand on Activated Platelets Triggers an Inflammatory Reaction of Endothelial Cells,” Nature 391, no. 6667 (1998): 591–594, 10.1038/35393. 9468137

[11] E. Lindmark , T. Tenno , and A. Siegbahn , “Role of Platelet P‐Selectin and CD40 Ligand in the Induction of Monocytic Tissue Factor Expression,” Arteriosclerosis, Thrombosis, and Vascular Biology 20, no. 10 (2000): 2322–2328, 10.1161/01.atv.20.10.2322. 11031222

[12] Y. Q. Ma , E. F. Plow , and J. G. Geng , “P‐Selectin Binding to P‐Selectin Glycoprotein Ligand‐1 Induces an Intermediate State of alphaMbeta2 Activation and Acts Cooperatively With Extracellular Stimuli to Support Maximal Adhesion of Human Neutrophils,” Blood 104, no. 8 (2004): 2549–2556, 10.1182/blood-2004-03-1108. 15217824

[13] P. A. d. C. Martins , J. M. van Gils , A. Mol , P. L. Hordijk , and J. J. Zwaginga , “Platelet Binding to Monocytes Increases the Adhesive Properties of Monocytes by Up‐Regulating the Expression and Functionality of β1 and β2 Integrins,” Journal of Leukocyte Biology 79, no. 3 (2006): 499–507, 10.1189/jlb.0605318. 29350799

[14] E. D. Hottz , I. M. Medeiros‐de‐Moraes , A. Vieira‐de‐Abreu , et al., “Platelet Activation and Apoptosis Modulate Monocyte Inflammatory Responses in Dengue,” Journal of Immunology 193, no. 4 (2014): 1864–1872, 10.4049/jimmunol.1400091. PMC4137323 25015827

[15] R. Polanowska‐Grabowska , K. Wallace , J. J. Field , et al., “P‐Selectin‐Mediated Platelet‐Neutrophil Aggregate Formation Activates Neutrophils in Mouse and Human Sickle Cell Disease,” Arteriosclerosis, Thrombosis, and Vascular Biology 30, no. 12 (2010): 12, 10.1161/ATVBAHA.110.211615. PMC3058044 21071696

[16] A. L. Frelinger, 3rd , J. A. Jakubowski , J. K. Brooks , et al., “Platelet Activation and Inhibition in Sickle Cell Disease (Pains) Study,” Platelets 25, no. 1 (2014): 1, 10.3109/09537104.2013.770136. 23469943

[17] H. A. Brittain , J. R. Eckman , R. A. Swerlick , R. J. Howard , and T. M. Wick , “Thrombospondin From Activated Platelets Promotes Sickle Erythrocyte Adherence to Human Microvascular Endothelium Under Physiologic Flow: A Potential Role for Platelet Activation in Sickle Cell Vaso‐Occlusion,” Blood 81, no. 8 (1993): 8. 8471771

[18] V. M. Dominical , L. Samsel , J. S. Nichols , et al., “Prominent Role of Platelets in the Formation of Circulating Neutrophil‐Red Cell Heterocellular Aggregates in Sickle Cell Anemia,” Haematologica 99, no. 11 (2014): e214–e217, 10.3324/haematol.2014.108555. PMC4222482 25420284

[19] R. Proença‐Ferreira , A. F. Brugnerotto , V. T. Garrido , et al., “Endothelial Activation by Platelets From Sickle Cell Anemia Patients,” PLoS One 9, no. 2 (2014): e89012, 10.1371/journal.pone.0089012. PMC3923877 24551209

[20] D. Zhang , C. Xu , D. Manwani , and P. S. Frenette , “Neutrophils, Platelets, and Inflammatory Pathways at the Nexus of Sickle Cell Disease Pathophysiology,” Blood 127, no. 7 (2016): 7, 10.1182/blood-2015-09-618538. PMC4760086 26758915

[21] J. Davila , D. Manwani , L. Vasovic , et al., “A Novel Inflammatory Role for Platelets in Sickle Cell Disease,” Platelets 26, no. 8 (2015): 8, 10.3109/09537104.2014.983891. 25548984

[22] K. V. Swanson , M. Deng , and J. P. Y. Ting , “The NLRP3 Inflammasome: Molecular Activation and Regulation to Therapeutics,” Nature Reviews Immunology 19, no. 8 (2019): 477–489, 10.1038/s41577-019-0165-0. PMC7807242 31036962

[23] E. D. Hottz , J. F. Lopes , C. Freitas , et al., “Platelets Mediate Increased Endothelium Permeability in Dengue Through NLRP3‐Inflammasome Activation,” Blood 122, no. 20 (2013): 20, 10.1182/blood-2013-05-504449. PMC3829114 24009231

[24] P. Murthy , F. Durco , J. L. Miller‐Ocuin , et al., “The NLRP3 Inflammasome and Bruton's Tyrosine Kinase in Platelets Co‐Regulate Platelet Activation, Aggregation, and In Vitro Thrombus Formation,” Biochemical and Biophysical Research Communications 483, no. 1 (2017): 1, 10.1016/j.bbrc.2016.12.161. 28034752

[25] J. Villagra , S. Shiva , L. A. Hunter , R. F. Machado , M. T. Gladwin , and G. J. Kato , “Platelet Activation in Patients With Sickle Disease, Hemolysis‐Associated Pulmonary Hypertension, and Nitric Oxide Scavenging by Cell‐Free Hemoglobin,” Blood 110, no. 6 (2007): 6, 10.1182/blood-2006-12-061697. PMC1976348 17536019

[26] C. C. Helms , M. Marvel , W. Zhao , et al., “Mechanisms of Hemolysis‐Associated Platelet Activation,” Journal of Thrombosis and Haemostasis 11, no. 12 (2013): 12, 10.1111/jth.12422. PMC3947421 24119131

[27] R. Singhal , G. K. Annarapu , A. Pandey , et al., “Hemoglobin Interaction With GP1balpha Induces Platelet Activation and Apoptosis: A Novel Mechanism Associated With Intravascular Hemolysis,” Haematologica 100, no. 12 (2015): 12, 10.3324/haematol.2015.132183. PMC4666328 26341739

[28] F. F. Dutra , L. S. Alves , D. Rodrigues , et al., “Hemolysis‐Induced Lethality Involves Inflammasome Activation by Heme,” Proceedings of the National Academy of Sciences of the United States of America 111, no. 39 (2014): 39, 10.1073/pnas.1405023111. PMC4191786 25225402

[29] D. Schaale , Z. Laspa , A. Balmes , et al., “Hemin Promotes Platelet Activation and Plasma Membrane Disintegration Regulated by the Subtilisin‐Like Proprotein Convertase Furin,” FASEB Journal 38, no. 21 (2024): e70155, 10.1096/fj.202400863RR. 39530531

[30] S. Vogel , T. Arora , X. Wang , et al., “The Platelet NLRP3 Inflammasome Is Upregulated in Sickle Cell Disease via HMGB1/TLR4 and Bruton Tyrosine Kinase,” Blood Advances 2, no. 20 (2018): 20, 10.1182/bloodadvances.2018021709. PMC6199654 30333099

[31] B. B. Nyakundi , A. Tóth , E. Balogh , et al., “Oxidized Hemoglobin Forms Contribute to NLRP3 Inflammasome‐Driven IL‐1β Production Upon Intravascular Hemolysis,” Biochimica et Biophysica Acta, Molecular Basis of Disease 1865, no. 2 (2019): 464–475, 10.1016/j.bbadis.2018.10.030. 30389578

[32] C. D. Reiter , X. Wang , J. E. Tanus‐Santos , et al., “Cell‐Free Hemoglobin Limits Nitric Oxide Bioavailability in Sickle‐Cell Disease,” Nature Medicine 8, no. 12 (2002): 12, 10.1038/nm799. 12426562

[33] M. Vissa , S. K. Larkin , E. P. Vichinsky , F. A. Kuypers , and E. Soupene , “Assessment of Total and Unbound Cell‐Free Heme in Plasma of Patients With Sickle Cell Disease,” Experimental Biology and Medicine 248, no. 10 (2023): 897–907, 10.1177/15353702231157920. PMC10484191 36941786

[34] N. Conran and E. V. De Paula , “Thromboinflammatory Mechanisms in Sickle Cell Disease ‐ Challenging the Hemostatic Balance,” Haematologica 105, no. 10 (2020): 2380–2390, 10.3324/haematol.2019.239343. PMC7556678 33054078

[35] S. R. Clark , A. C. Ma , S. A. Tavener , et al., “Platelet TLR4 Activates Neutrophil Extracellular Traps to Ensnare Bacteria in Septic Blood,” Nature Medicine 13, no. 4 (2007): 463–469, 10.1038/nm1565. 17384648

[36] T. Wun , T. Paglieroni , F. Tablin , J. Welborn , K. Nelson , and A. Cheung , “Platelet Activation and Platelet‐Erythrocyte Aggregates in Patients With Sickle Cell Anemia,” Journal of Laboratory and Clinical Medicine 129, no. 5 (1997): 507–516. 10.1016/s0022-2143(97)90005-6 9142047

[37] D. P. Inwald , F. J. Kirkham , M. J. Peters , et al., “Platelet and Leucocyte Activation in Childhood Sickle Cell Disease: Association With Nocturnal Hypoxaemia,” British Journal of Haematology 111, no. 2 (2000): 474–481. 10.1046/j.1365-2141.2000.02353.x 11122087

[38] A. Zarbock , R. K. Polanowska‐Grabowska , and K. Ley , “Platelet‐Neutrophil‐Interactions: Linking Hemostasis and Inflammation,” Blood Reviews 21, no. 2 (2007): 99–111, 10.1016/j.blre.2006.06.001. 16987572

[39] É. M. F. Gotardo , L. S. Torres , B. C. Zaidan , et al., “Targeting P‐Selectin and Interleukin‐1β in Mice With Sickle Cell Disease: Effects on Vaso‐Occlusion, Liver Injury and Organ Iron Deposition,” Haematologica 110, no. 3 (2025): 725–738, 10.3324/haematol.2024.286418. PMC11873698 39568425

[40] B. Z. Shao , Z. Q. Xu , B. Z. Han , D. F. Su , and C. Liu , “NLRP3 Inflammasome and Its Inhibitors: A Review,” Frontiers in Pharmacology 6 (2015): 262, 10.3389/fphar.2015.00262. PMC4633676 26594174

[41] N. Conran and J. D. Belcher , “Inflammation in Sickle Cell Disease,” Clinical Hemorheology and Microcirculation 68, no. 2–3 (2018): 263–299, 10.3233/CH-189012. PMC6314308 29614637

[42] D. C. Rees , Y. Kilinc , S. Unal , et al., “A Randomized, Placebo‐Controlled, Double‐Blind Trial of Canakinumab in Children and Young Adults With Sickle Cell Anemia,” Blood 139, no. 17 (2022): 2642–2652, 10.1182/blood.2021013674. 35226723

[43] M. Ito , T. Shichita , M. Okada , et al., “Bruton's Tyrosine Kinase Is Essential for NLRP3 Inflammasome Activation and Contributes to Ischaemic Brain Injury,” Nature Communications 6 (2015): 7360, 10.1038/ncomms8360. PMC4490404 26059659

[44] X. Liu , T. Pichulik , O. O. Wolz , et al., “Human NACHT, LRR, and PYD Domain‐Containing Protein 3 (NLRP3) Inflammasome Activity Is Regulated by and Potentially Targetable Through Bruton Tyrosine Kinase,” Journal of Allergy and Clinical Immunology 140, no. 4 (2017): 4, 10.1016/j.jaci.2017.01.017. 28216434

[45] N. Cardenes , C. Corey , L. Geary , et al., “Platelet Bioenergetic Screen in Sickle Cell Patients Reveals Mitochondrial Complex V Inhibition, Which Contributes to Platelet Activation,” Blood 123, no. 18 (2014): 2864–2872, 10.1182/blood-2013-09-529420. PMC4007612 24677541

[46] G. K. Annarapu , R. Singhal , A. Gupta , et al., “HbS Binding to GP1bα Activates Platelets in Sickle Cell Disease,” PLoS One 11, no. 12 (2016): e0167899, 10.1371/journal.pone.0167899. PMC5148012 27936141

[47] J. H. Bourne , M. Colicchia , Y. Di , et al., “Heme Induces Human and Mouse Platelet Activation Through C‐Type‐Lectin‐Like Receptor‐2,” Haematologica 106, no. 2 (2021): 626–629, 10.3324/haematol.2020.246488. PMC7849553 32354867

[48] S. Vogel , R. Bodenstein , Q. Chen , et al., “Platelet‐Derived HMGB1 Is a Critical Mediator of Thrombosis,” Journal of Clinical Investigation 125, no. 12 (2015): 4638–4654, 10.1172/JCI81660. PMC4665785 26551681

[49] W. Chi , H. Chen , F. Li , Y. Zhu , W. Yin , and Y. Zhuo , “HMGB1 Promotes the Activation of NLRP3 and Caspase‐8 Inflammasomes via NF‐kappaB Pathway in Acute Glaucoma,” Journal of Neuroinflammation 12 (2015): 137, 10.1186/s12974-015-0360-2. PMC4518626 26224068

[50] S. Charache , G. J. Dover , R. D. Moore , et al., “Hydroxyurea: Effects on Hemoglobin F Production in Patients With Sickle Cell Anemia,” Blood 79, no. 10 (1992): 2555–2565. 1375104

[51] J. Erdei , A. Tóth , E. Balogh , et al., “Induction of NLRP3 Inflammasome Activation by Heme in Human Endothelial Cells,” Oxidative Medicine and Cellular Longevity 2018 (2018): 4310816, 10.1155/2018/4310816. PMC5883980 29743981

[52] S. K. NaveenKumar , M. Hemshekhar , K. Kemparaju , and K. S. Girish , “Hemin‐Induced Platelet Activation and Ferroptosis Is Mediated Through ROS‐Driven Proteasomal Activity and Inflammasome Activation: Protection by Melatonin,” Biochimica et Biophysica Acta, Molecular Basis of Disease 1865, no. 9 (2019): 2303–2316, 10.1016/j.bbadis.2019.05.009. 31102787

[53] Z. Li , M. K. Delaney , K. A. O'Brien , and X. Du , “Signaling During Platelet Adhesion and Activation,” Arteriosclerosis, Thrombosis, and Vascular Biology 30, no. 12 (2010): 2341–2349, 10.1161/ATVBAHA.110.207522. PMC3085271 21071698

[54] J. Liu , M. E. Fitzgerald , M. C. Berndt , C. W. Jackson , and T. K. Gartner , “Bruton Tyrosine Kinase Is Essential for Botrocetin/VWF‐Induced Signaling and GPIb‐Dependent Thrombus Formation In Vivo,” Blood 108, no. 8 (2006): 2596–2603, 10.1182/blood-2006-01-011817. PMC1895591 16788103

[55] P. L. R. Nicolson , S. H. Nock , J. Hinds , et al., “Low‐Dose Btk Inhibitors Selectively Block Platelet Activation by CLEC‐2,” Haematologica 106, no. 1 (2021): 208–219, 10.3324/haematol.2019.218545. PMC7776357 31949019

[56] R. Zhou , A. S. Yazdi , P. Menu , and J. Tschopp , “A Role for Mitochondria in NLRP3 Inflammasome Activation,” Nature 469, no. 7329 (2011): 221–225, 10.1038/nature09663. 21124315

[57] M. E. Heid , P. A. Keyel , C. Kamga , S. Shiva , S. C. Watkins , and R. D. Salter , “Mitochondrial Reactive Oxygen Species Induces NLRP3‐Dependent Lysosomal Damage and Inflammasome Activation,” Journal of Immunology 191, no. 10 (2013): 5230–5238, 10.4049/jimmunol.1301490. PMC3833073 24089192

[58] S. Alfonso‐Loeches , J. R. Ureña‐Peralta , M. J. Morillo‐Bargues , J. Oliver‐De La Cruz , and C. Guerri , “Role of Mitochondria ROS Generation in Ethanol‐Induced NLRP3 Inflammasome Activation and Cell Death in Astroglial Cells,” Frontiers in Cellular Neuroscience 8 (2014): 216, 10.3389/fncel.2014.00216. PMC4118026 25136295

[59] R. Vats , T. Brzoska , M. F. Bennewitz , et al., “Platelet Extracellular Vesicles Drive Inflammasome‐IL‐1β‐Dependent Lung Injury in Sickle Cell Disease,” American Journal of Respiratory and Critical Care Medicine 201, no. 1 (2020): 33–46, 10.1164/rccm.201807-1370OC. PMC6938158 31498653

[60] Sanofi , “A 52‐Week, Multicenter, Randomized, Double‐Blind, Placebo‐Controlled, Parallel‐Group, Group Sequential Study to Evaluate the Efficacy and Safety of Rilzabrutinib in Patients Aged 10 to 65 Years With Sickle‐Cell Disease,” (2026).

