# Supplementary material for: Activation of Platelet Caspase‐1 and P‐Selectin Over‐Expression in Sickle Cell Disease Patients: An Involvement of NLRP3 Inflammasome?
Source: J Cell Mol Med. 2026 Oct 14;30(20):e71390. doi: 10.1111/jcmm.71390 (PMC13669531; doi:10.1111/jcmm.71390)
Supplement: Supplementary file 1 [file JCMM-30-e71390-s001.pdf]

SUPPLEMENTARY DATA

Figure S1. Platelet activation in SS patients treated and untreated with hydroxycarbamide.

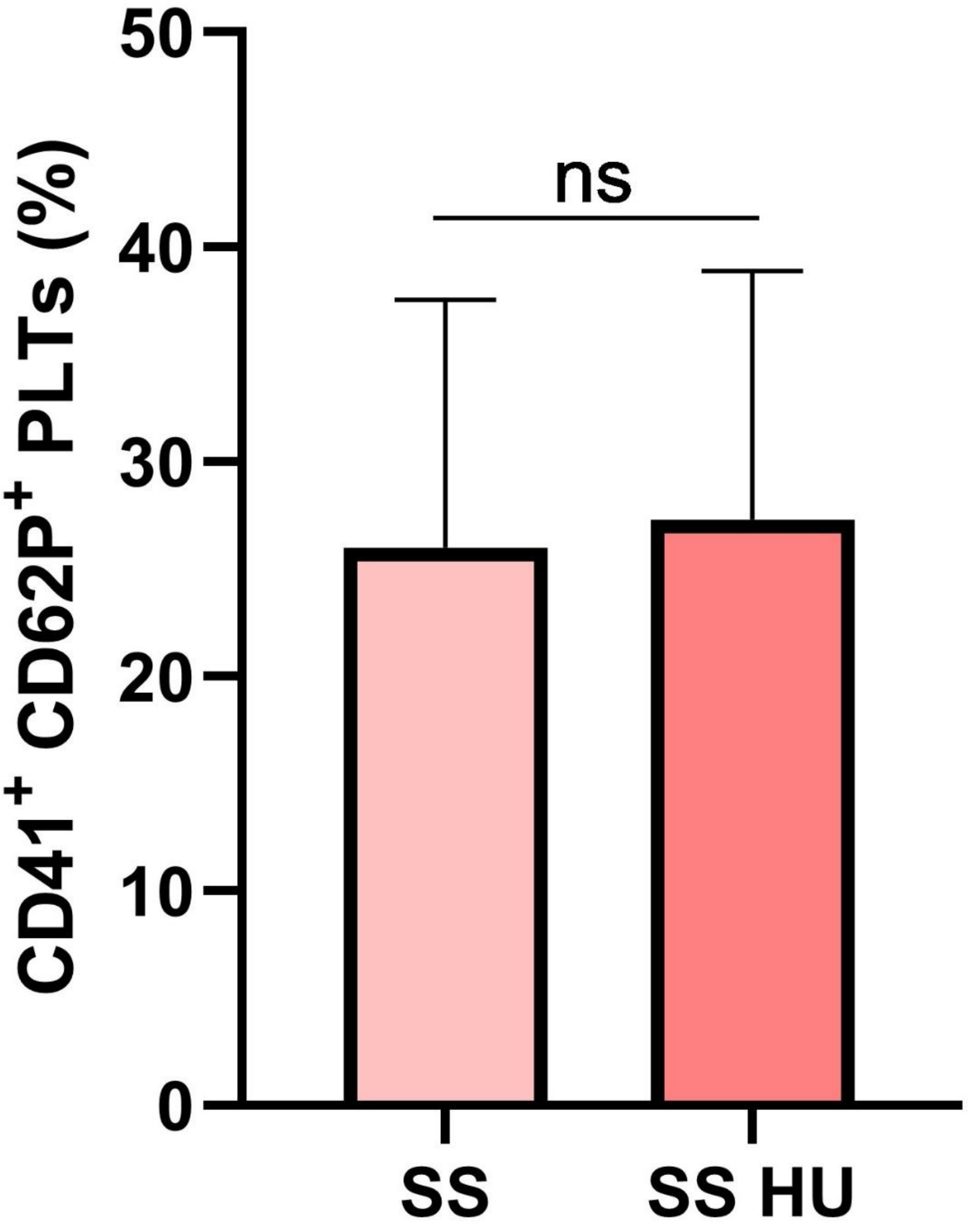

Table S1. Clinical characteristics and platelet activation markers of studied patients.

	**Non-VOC**	**VOC**	***p* values**
**$CD41^+CD62P^+$ PLTs, mean (± SD), %**	25.9 (10.2)	25.1 (12.6)	0.7638
**$CD41^+FLICA^+$ PLTs, median (IQR), %**	8.7 (5.0-17.5)	9.2 (4.2-25.9)	0.9806
**PLT-PNN aggregates, median (IQR), %**	9.8 (7.5 -20.3)	14.1 (10.5-24.0)	0.1226
	**Non-ACS**	**ACS**	***p* values**
**$CD41^+CD62P^+$ PLTs, mean (± SD), %**	25.7 (11.4)	26.83 (5.7)	0.7779
**$CD41^+FLICA^+$ PLTs, median (IQR), %**	7.9 (4.4-17.5)	14.8 (6.1-27.0)	0.2111
**PLT-PNN aggregates, median (IQR), %**	11.5 (7.9-20.2)	16.91 (9.5-30.2)	0.2678

Figure S2. Platelet-neutrophil aggregates in HC treated and untreated SS patients.

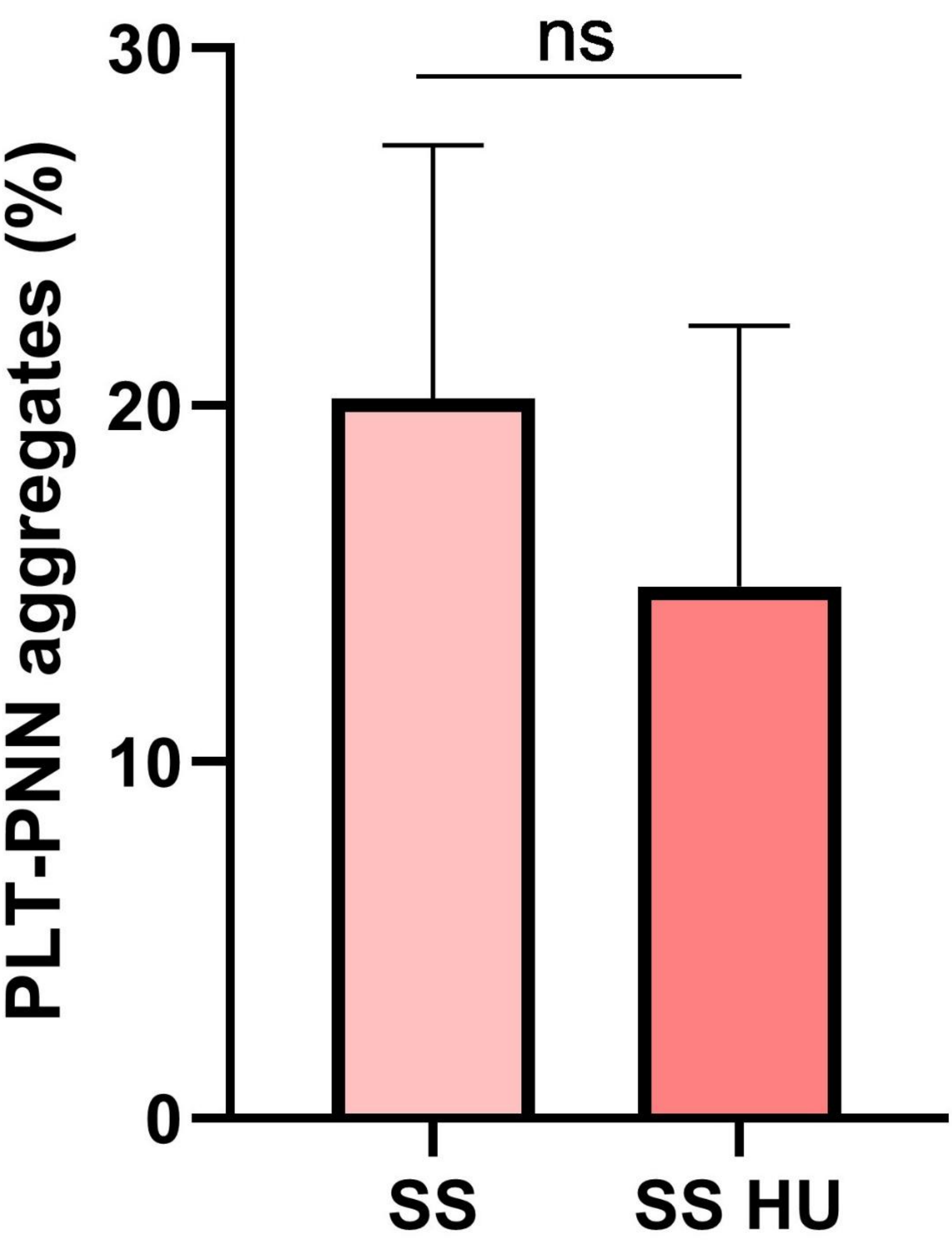

Figure S3. Caspase-1 activation in HC treated and untreated SS patients.

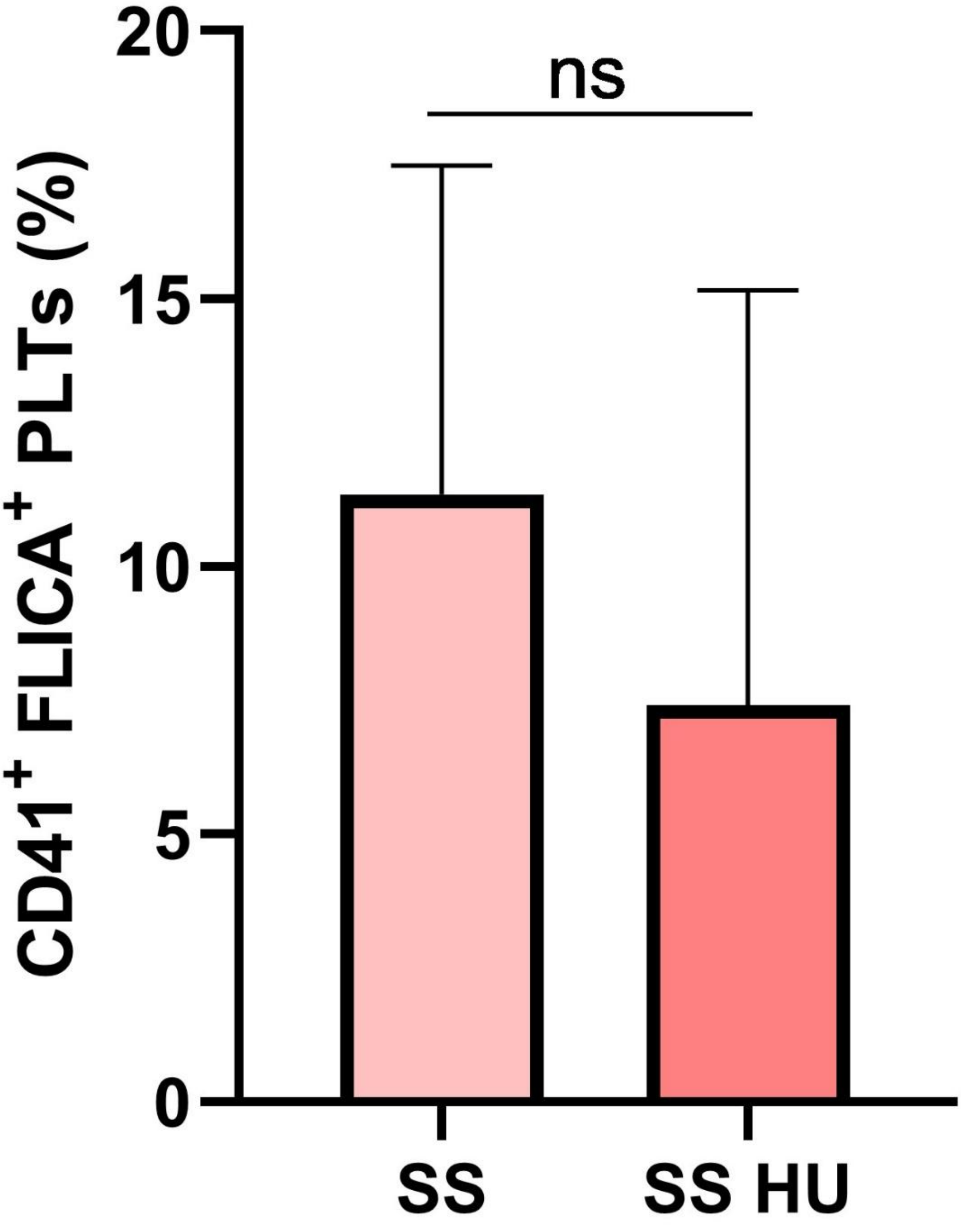

Table S2. Correlation between rate of CD41^{+} FLICA^{+} platelets and hemolysis/anemia markers in sickle cell patients.

	SS patients			SC patients		
	**Pearson *r***	**95% CI**	***p* values**	**Pearson *r***	**95% CI**	***p* values**
**CD41^{+} FLICA^{+} PLTs (%)**						
RET (%)	0.2825	-0.01575 to 0.5345	0.0632	-0.1192	-0.4443 to 02337	0.5087
HbF (%)	-0.3872	-0.6381 to -0.06198	**0.0216**	0.04723	-0.5413 to 0.6047	0.8841
Total bilirubin (µM)	0.3238	0.02978 to 0.5662	**0.0320**	-0.1643	-0.4852 to 0,1956	0.3688
ASAT (U/L)	0.2596	-0.04044 to 0.5166	0.0889	-0.04811	-0.3903 to 0.3057	0.7937

Figure S4. Platelet activation in SS and SC patients correlate with platelet caspase-1 activation.

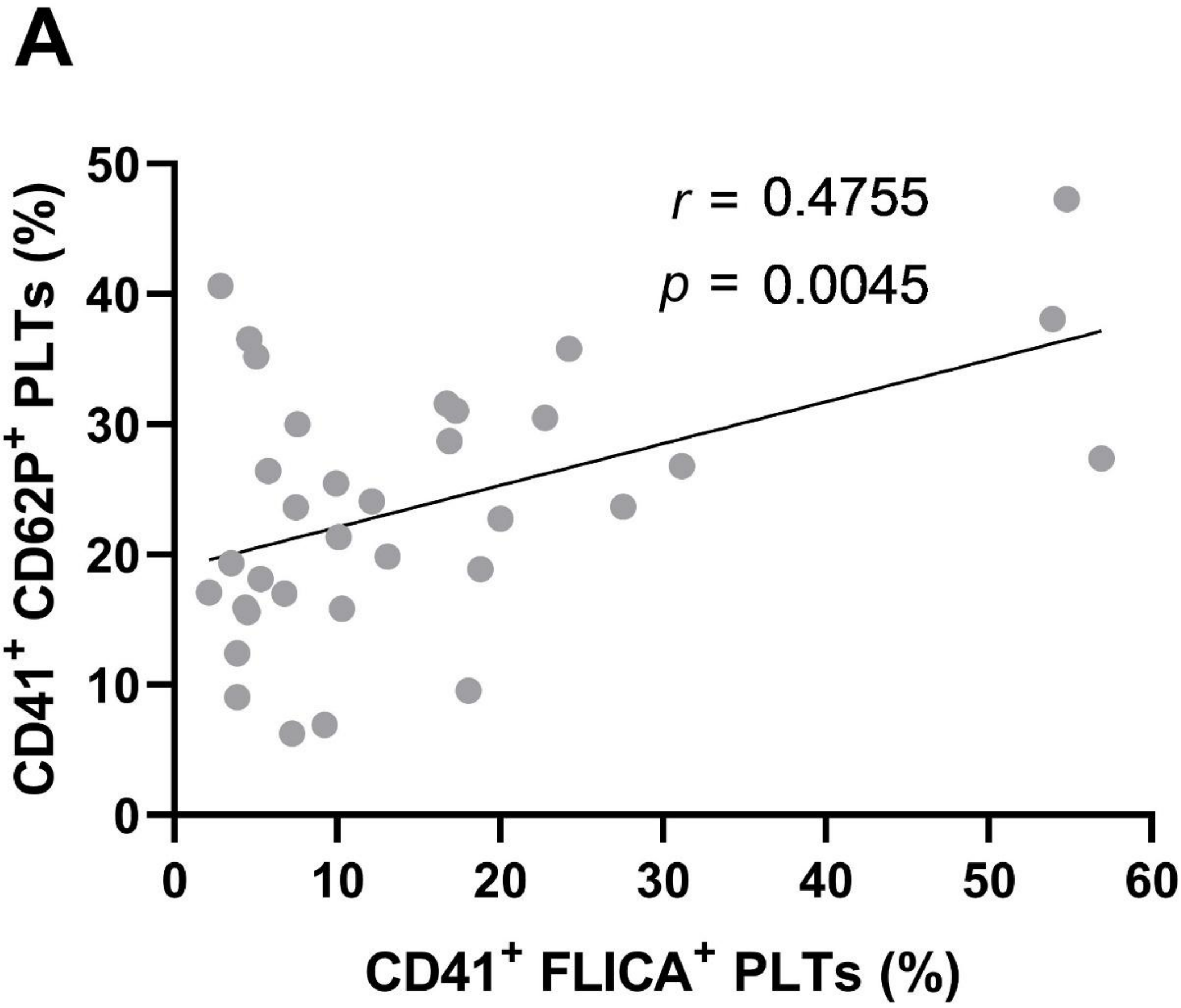

B

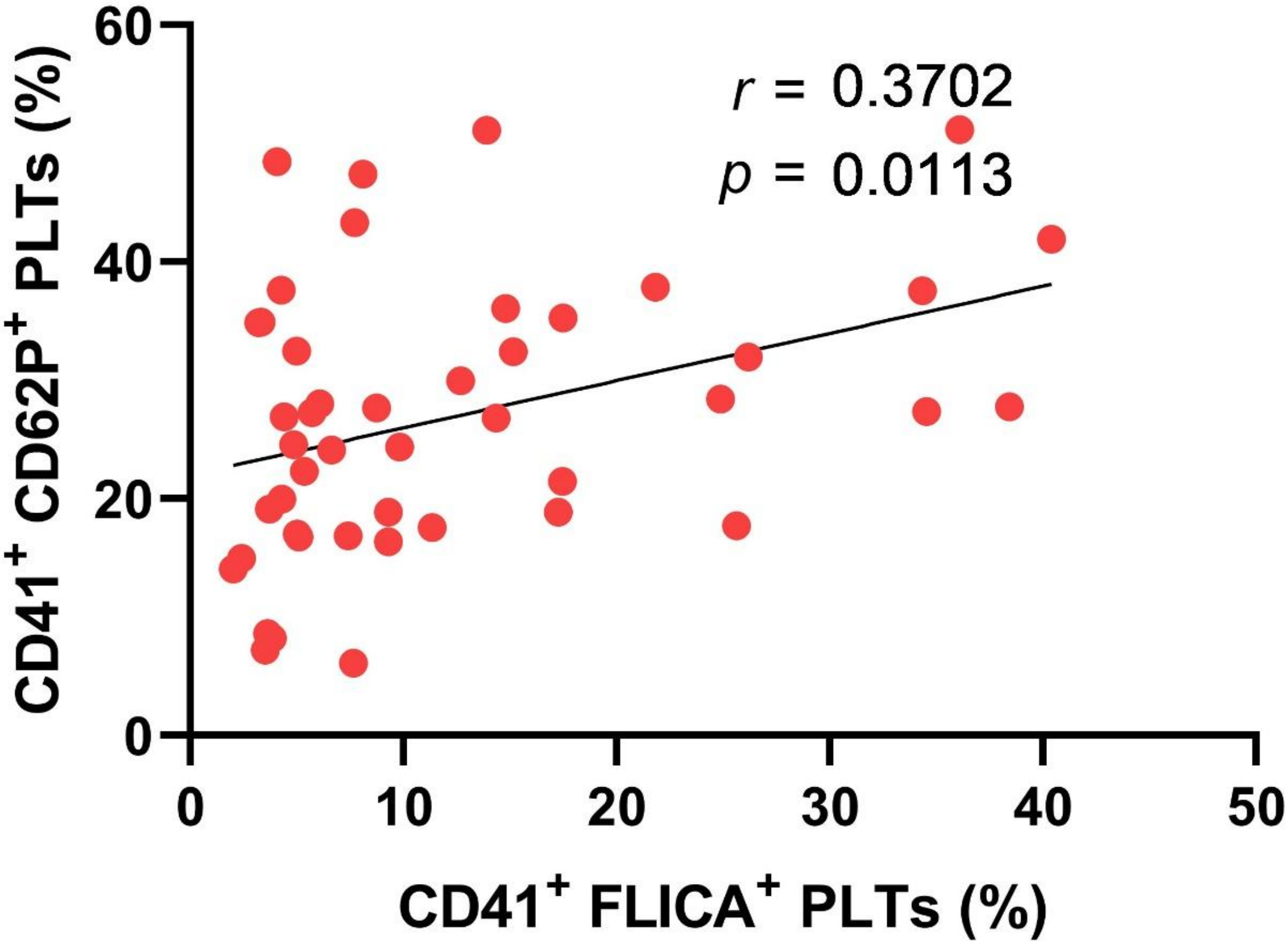

r = 0.3702
p = 0.0113
CD41+ CD62P+ PLTs (%)
CD41+ FLICA+ PLTs (%)
0
20
40
60
0
10
20
30
40
50
